# Real-World Clinical Outcomes of Biosimilar Trastuzumab (CT-P6) in HER2-Positive Early-Stage and Metastatic Breast Cancer

**DOI:** 10.3389/fonc.2021.689587

**Published:** 2021-06-04

**Authors:** Soong June Bae, Jee Hung Kim, Sung Gwe Ahn, Hei-Cheul Jeung, Joohyuk Sohn, Gun Min Kim, Min Hwan Kim, Seung Il Kim, Seho Park, Hyung Seok Park, Ji Ye Kim, Joon Jeong

**Affiliations:** ^1^ Department of Surgery, Gangnam Severance Hospital, Yonsei University College of Medicine, Seoul, South Korea; ^2^ Institute for Breast Cancer Precision Medicine, Yonsei University College of Medicine, Seoul, South Korea; ^3^ Division of Medical Oncology, Department of Internal Medicine, Gangnam Severance Hospital, Yonsei University College of Medicine, Seoul, South Korea; ^4^ Division of Medical Oncology, Department of Internal Medicine, Yonsei Cancer Center, Yonsei University College of Medicine, Seoul, South Korea; ^5^ Department of Surgery, Yonsei Cancer Center, Yonsei University College of Medicine, Seoul, South Korea

**Keywords:** biosimilar, HER2-positive breast cancer, trastuzumab, CT-P6, early breast cancer (EBC), metastatic breast cancer (mbc)

## Abstract

**Background:**

The trastuzumab biosimilar CT-P6 has demonstrated equivalent efficacy and comparable safety to reference trastuzumab (RTZ) in clinical trials of human epidermal growth factor receptor 2 (HER2)-positive early breast cancer (EBC). Here, we present the first real-world comparison of CT-P6 versus RTZ with dual HER2-targeted therapy for the neoadjuvant and palliative first-line treatment with HER2-positive EBC and metastatic breast cancer (MBC) patients in two tertiary hospitals in Korea.

**Methods:**

We retrospectively investigated medical records in the Severance Breast Cancer Registry in Korea. We identified patients with HER2-positive EBC (n=254) who had received neoadjuvant chemotherapy with RTZ or CT-P6, plus pertuzumab, carboplatin and docetaxel (TCHP) and untreated stage IV MBC (n=103) who had received palliative first-line treatment with RTZ or CT-P6, plus pertuzumab and docetaxel (THP) between May 2014 and December 2019. The primary endpoints were pathologic complete response (pCR) in the EBC and progression-free survival (PFS) in the MBC cohort. Overall survival (OS), overall response rate (ORR), disease control rate (DCR), and cardiac safety were secondary endpoints.

**Results:**

A similar percentage of EBC patients achieved a pCR with CT-P6 versus RTZ (74.4% [93/125]) vs 69.8% [90/129], *p*=0.411). For patients with MBC, median follow-up duration was 23.0 and 41.0 months for CT-P6 and RTZ groups, respectively; median PFS did not differ significantly between two groups (13.0 vs 18.0 months, 95% confidence intervals (CIs) 0.0-26.6 vs 11.3-24.7, *p*=0.976). The ORR, DCR, and cardiac safety profiles did not also show significant difference efficacy outcomes between two groups.

**Conclusions:**

These real-world data suggest that biosimilar trastuzumab CT-P6 has similar effectiveness and cardiac safety to RTZ in HER2-positive EBC and MBC patients, when administered as part of dual HER2-targeted therapy with pertuzumab plus chemotherapy in the neoadjuvant or palliative setting.

## Introduction

Breast cancer is a heterogeneous disease that has multiple subtypes. Approximately 25% of breast cancers amplify the human epidermal growth factor receptor 2 (HER2) oncogene, resulting in a more aggressive phenotype with poorer prognosis (1). The development of trastuzumab, a humanized monoclonal antibody that binds to the HER2 extracellular domain, has transformed the treatment of HER2-positive breast cancers (2) and improved clinical responses (2–4) and disease free survival (5). Trastuzumab is also effective in metastatic HER2-positive breast cancer, with the addition of trastuzumab to standard chemotherapy shown to extend the overall survival (OS) (6, 7).

However, the development of novel biologic agents is expensive, which can translate into high drug costs (8). Despite their efficacy, the costs associated with these drugs can pose a burden on healthcare systems and create barriers to access. This difficulty can be mitigated in part through the use of biosimilars (8). A biosimilar is a drug that is highly similar to an existing drug, the originator or reference product, and which shows no clinically meaningful differences in purity, safety, and efficacy (8). Biosimilars are in general more affordable than their reference products, and their availability has the potential to improve patient access to safe and effective treatments.

Several biosimilars of trastuzumab have been developed, including CT-P6 (Herzuma^®^; Celltrion Inc., Incheon, Republic of Korea) (9, 10), SB3 (Ontruzant^®^; Samsung Bioepis Co., Ltd), (11) ABP 980 (Kanjinti^®^; Amgen) (12) and PF-05280014 (Trazimera^®^; Pfizer) (13). CT-P6 binds with high affinity and specificity to the same HER2 epitope as the reference product, Herceptin^®^ (Genentech) (9, 10). In 2018, CT-P6 was approved by the US Food and Drug Administration and the European Medicines Agency for the treatment of HER2-positive early (EBC) and metastatic breast cancer (MBC) (14–17).

Real-world studies are an important supplement to clinical trials, by revealing the long-term safety and effectiveness of drugs in broader patient populations, as well as in other settings and in combination with other treatments. For example, reference trastuzumab (RTZ) is now increasingly used in combination with pertuzumab (Perjeta^®^; Genentech) as part of dual HER2-targeted therapy. Pertuzumab is a monoclonal antibody that targets a different region of the HER2 receptor to trastuzumab, (18) and thus has a complementary mode of action. Dual HER2-targeting with trastuzumab and pertuzumab, plus chemotherapy, has been shown to improve clinical responses compared to trastuzumab alone plus chemotherapy in HER2-positive EBC (19, 20) and MBC (21).

Here we present the results of the first real-world comparison of the effectiveness of CT-P6 and RTZ when administered with pertuzumab plus chemotherapy in the neoadjuvant treatment to patients with HER2-positive EBC or in the palliative first-line treatment to patients with MBC. As cardiotoxicity is a potentially serious adverse effect associated with the use of trastuzumab, (6) we also assessed the cardiac safety of CT-P6 and the RTZ.

## Materials and Methods

### Patients

**Figure 1:**
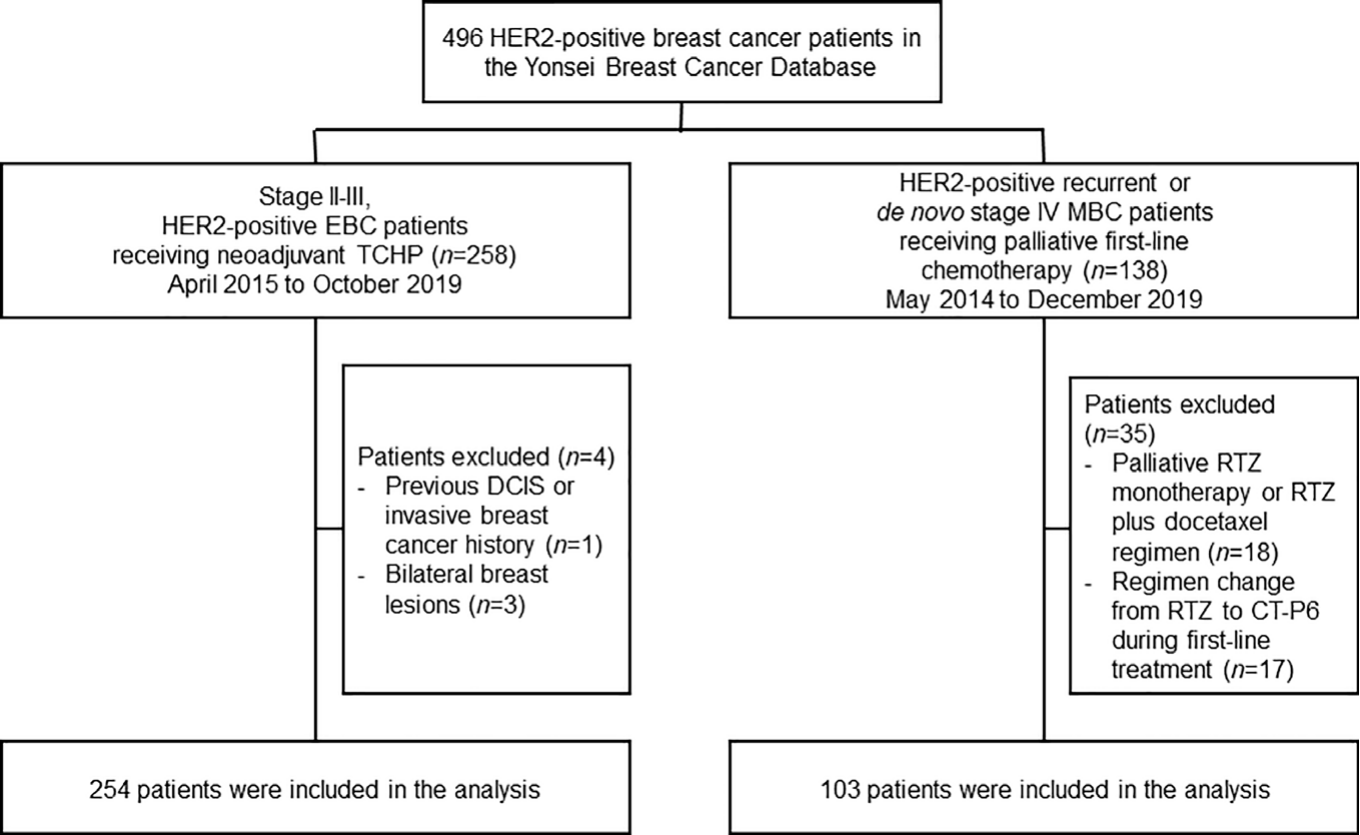
Study profile. DCIS, ductal carcinoma in situ; EBC, early-stage breast cancer; HER2, human epidermal growth factor receptor 2; MBC, metastatic breast cancer; RTZ, reference trastuzumab; TCHP, docetaxel–carboplatin–RTZ or CT-P6–pertuzumab.

We retrospectively investigated medical records in the Severance Breast Cancer Registry at the Yonsei Cancer Center and Gangnam Severance Hospital in Seoul, Republic of Korea (**Figure 1**). We identified patients with HER2-positive EBC who had undergone neoadjuvant chemotherapy between April 2015 and October 2019, as well as patients with newly diagnosed *de novo* or recurrent HER2-positive MBC who had undergone palliative chemotherapy between May 2014 and December 2019. For both the EBC and MBC cohorts, eligible patients were women aged >19 years with a histologically confirmed diagnosis of HER2-positive breast cancer.

EBC was defined as clinical stage II–III, classified according to the American Joint Committee on Cancer Breast Cancer Staging seventh edition. In MBC cohort, eligible patients had no previous palliative treatment in advanced disease and received chemotherapy with or without trastuzumab in the neoadjuvant or adjuvant setting, provided there was a minimum 12-month interval between completion of all therapy and the diagnosis of metastatic disease.

### Procedures

In the EBC cohort, neoadjuvant docetaxel (T)-carboplatin (C)-RTZ or CT-P6 (H)-pertuzumab (P) (TCHP) treatment was administered *via* intravenous infusion every 3 weeks, for a total of six cycles, in accordance with procedures in the TRYPHAENA trial (22). CT-P6 or RTZ were administered at a loading dose of 8 mg/kg on cycle 1, and at a maintenance dose of 6 mg/kg on cycles 2–6. Pertuzumab was administered at a loading dose of 840 mg, then a maintenance dose of 420 mg in subsequent cycles. Carboplatin was administered at area under the curve 5 or 6 and docetaxel at 75 mg/m^2^, in all cycles.

In the MBC cohort, palliative first-line docetaxel (T)-RTZ or CT-P6 (H)-pertuzumab (P) (THP) regimen was administered *via* intravenous infusion every 3 weeks, in accordance with procedures in the CLEOPATRA trial (23). Pertuzumab, docetaxel and RTZ or CT-P6 were given same doses and schedules in neoadjuvant setting. Pertuzumab and RTZ or CT-P6 were given until disease progression or unmanageable toxic effects, and docetaxel was given for at least six cycles. If chemotherapy was discontinued due to toxic effects, pertuzumab and RTZ or CT-P6 were continued until disease progression, or occurrence of unacceptable adverse events.

### Outcome Measures

The primary endpoints were pathologic complete response (pCR) in the EBC and progression-free survival (PFS) in the MBC cohort. pCR was assessed locally at the time of surgery and was defined as the absence of invasive tumor cells during a microscopic assessment of the primary tumor (ypT0/is) and axilla (ypN0). Median PFS was defined as time from the date of first-line palliative systemic treatment to the first documented disease progression or death from any cause.

The secondary endpoints in the MBC cohort were median overall survival (mOS), overall response rate (ORR) and disease control rate (DCR). mOS was the time from the date of first-line palliative systemic treatment to death from any cause. ORR was defined as the proportion of patients who achieved a complete response (CR) or confirmed partial response (cPR) per RECIST 1.1. DCR was defined as the proportion of patients who achieved a CR, cPR, or stable disease (SD) per RECIST 1.1.

Cardiac safety was a secondary endpoint in both the EBC and MBC cohorts. Left ventricular ejection fraction (LVEF) was measured either by echocardiography or a multiple-gated acquisition (MUGA) scan every 3 months. An adverse event related to cardiac safety was defined as a decline in investigator-assessed LVEF of ≥10 percentage points from baseline at any time, or an LVEF of <50% at any time.

### Statistical Analysis

pCR rate was compared between treatment groups using the chi-square test or Fisher’s exact test. To reduce baseline confounders between the CT-P6 group and RTZ group, one-to-one propensity score matching (PSM) was performed using the nearest-neighbor matching method. Variables entered into the PSM included age, histologic type, histologic grade, estrogen receptor, progesterone receptor, subgroup, Ki67, clinical tumor stage, and clinical node stage. PFS and OS were evaluated using unadjusted log-rank tests and Cox proportional-hazards models. Differences in ORR or DCR between the treatment groups were evaluated using an adjusted Fisher’s exact test. The 95% confidence intervals (CIs) for the ORRs were calculated using an asymptotic normal approximation. A two-tailed *P*-value <0.05 was considered statistically significant. The statistical analysis was performed using IBM SPSS Statistics for Windows, Version 23 software (IBM Corp. Armonk, NY, USA).

## Results

### Study Population of EBC and MBC Cohorts

A total of 258 women with HER2-positive EBC were identified who underwent neoadjuvant TCHP, followed by surgery (**Figure 1**). Four patients were excluded owing to the presence of bilateral breast lesions (*n*=3) or a history of DCIS/invasive breast cancer (*n*=1). The remaining 254 women were analyzed in this study. Baseline characteristics were similar between patients who received CT-P6 (*n*=125, 49.2%) and those receiving RTZ (*n*=129, 50.8%) (**Table 1**). A greater proportion of patients in the CT-P6 had a Ki67 index ≥14 (CT-P6: 87.5% [84/125]; RTZ: 70.5% [55/129]; *P*=0.005). After adjusting for propensity scores, all variables were well balanced between the CT-P6 and RTZ groups (**Supplementary Table 1**).

**Table 1:** Baseline characteristics of patients with HER2-positive early-stage and metastatic breast cancer.

		Early-stage breast cancer				Metastatic breast cancer			
		RTZ (*n*=129)	CT-P6 (*n*=125)	Total (*N*=254)	*P*-value	RTZ (*n*=65)	CT-P6 (*n*=38)	Total (*N*=103)	*P*-value
Age, median (range)		49 (22–75)	50 (27–71)	49 (22–75)	0.447	54 (31–76)	55 (28–79)	54 (28–79)	0.695
Stage IV, *n* (%)									0.333
*De novo*		–	–	–		47 (72.3)	24 (63.2)	71 (68.9)	
Recurrent		–	–	–		18 (27.7)	14 (36.8)	32 (31.1)	
Histology, *n* (%)					<0.001				0.117
IDC		113 (87.6)	124 (99.2)	237 (93.3)		60 (93.8)	32 (86.5)	92 (91.1)	
ILC		11 (8.5)	1 (0.8)	12 (4.7)		1 (1.6)	0 (0)	1 (1.0)	
Other^a^		5 (3.9)	0	5 (2.0)		3 (4.7)	5 (13.5)	8 (7.9)	
ER, *n* (%)					0.752				0.804
Positive		44 (34.1)	45 (36.0)	89 (35.0)		29 (44.6)	16 (42.1)	45 (43.7)	
Negative		85 (65.9)	80 (64.0)	165 (65.0)		36 (55.4)	22 (57.9)	58 (56.3)	
PR, *n* (%)					0.992				0.812
Positive		30 (23.3)	29 (23.2)	59 (23.2)		15 (23.1)	8 (21.1)	23 (22.3)	
Negative		99 (76.7)	96 (76.8)	195 (76.8)		50 (76.9)	30 (78.9)	80 (77.7)	
Subgroup, *n* (%)					0.852				0.804
HR+/HER2+		45 (34.9)	45 (36.0)	90 (35.4)		29 (44.6)	16 (42.1)	45 (43.7)	
HR−/HER2+		84 (65.1)	80 (64.0)	164 (64.6)		36 (55.4)	22 (57.9)	58 (56.3)	
HG,^b^ *n* (%)					0.960				0.135
1 or 2		68 (73.1)	83 (72.8)	151 (72.9)		4 (36.4)	7 (70.0)	11 (52.4)	
3		25 (26.9)	31 (27.2)	56 (27.1)		7 (63.6)	3 (30.0)	10 (47.6)	
Ki67,^b^ *n* (%)					0.005				0.647
<14		23 (29.5)	12 (12.5)	35 (20.1)		3 (11.1)	3 (11.5)	6 (11.3)	
≥14		55 (70.5)	84 (87.5)	139 (79.9)		24 (88.9)	23 (88.5)	47 (88.7)	
cT,^c^ *n* (%)					0.139				
1 or 2		85 (65.9)	93 (74.4)	178 (70.1)		–	–	–	
≥3		44 (34.1)	32 (25.6)	76 (29.9)		–	–	–	
cN,^d^ *n* (%)					0.072				
Negative		16 (12.4)	26 (20.8)	42 (16.5)		–	–	–	
Positive		113 (87.6)	99 (79.2)	212 (83.5)		–	–	–	
Metastasis site, *n* (%)									
Distant LN		–	–	–		39 (60.0)	19 (50.0)	58 (56.3)	0.323
Bone		–	–	–		33 (50.8)	16 (42.1)	48 (47.6)	0.396
Lung		–	–	–		21 (32.3)	14 (36.8)	35 (34.0)	0.639
Liver		–	–	–		18 (29.2)	14 (36.8)	33 (32.0)	0.424
Breast skin		–	–	–		4 (6.2)	6 (15.8)	10 (9.7)	0.107
Brain		–	–	–		4 (6.2)	3 (7.9)	7 (6.8)	0.513
Pleural		–	–	–		3 (4.6)	4 (10.5)	7 (6.8)	0.225
Other^e^		–	–	–		9 (13.8)	2 (5.3)	11 (10.7)	0.151

a Other histology included micropapillary, apocrine, mucinous, and metaplastic carcinoma.

b Values were missing for some patients across groups.

c cT was based on tumor size measured by pre-treatment breast magnetic resonance imaging.

d cN positive was defined as axillary lymph node metastasis proven by fine-needle aspiration biopsy (FNAB), or suspicious axillary lymph node metastasis in the imaging study among patients who did not receive FNAB.

e Other metastasis sites included adrenal gland, peritoneum, and soft tissue mass.

cN, clinical node stage; cT, clinical tumor stage; ER, estrogen receptor; HER2, human epidermal growth factor receptor 2; HG, histologic grade; HR, hormone receptor; IDC, invasive ductal carcinoma; ILC, invasive lobular carcinoma; LN, lymph node; PR, progesterone receptor; RTZ, reference trastuzumab.

A total of 138 women with recurrent or *de novo* stage IV HER2-positive MBC were identified who received palliative first-line treatment with THP between May 2014 and December 2019. Eighteen patients were excluded because they received either palliative RTZ monotherapy or palliative RTZ plus docetaxel. A further 17 patients were excluded because they were switched from RTZ to CT-P6. The remaining 103 women were analyzed in this study. Baseline characteristics were similar for patients who received CT-P6 (*n*=38, 36.9%) and RTZ (*n*=65, 63.1%) (**Table 1**). The percentage of patients with *de novo* stage IV MBC was 63.2% (24/38) in the CT-P6 and 72.3% (47/65) among those who received RTZ. The most common sites of metastasis were distant lymph nodes, followed by bone, lung, and liver.

### Effectiveness of EBC and MBC Cohorts

**Figure 2:**
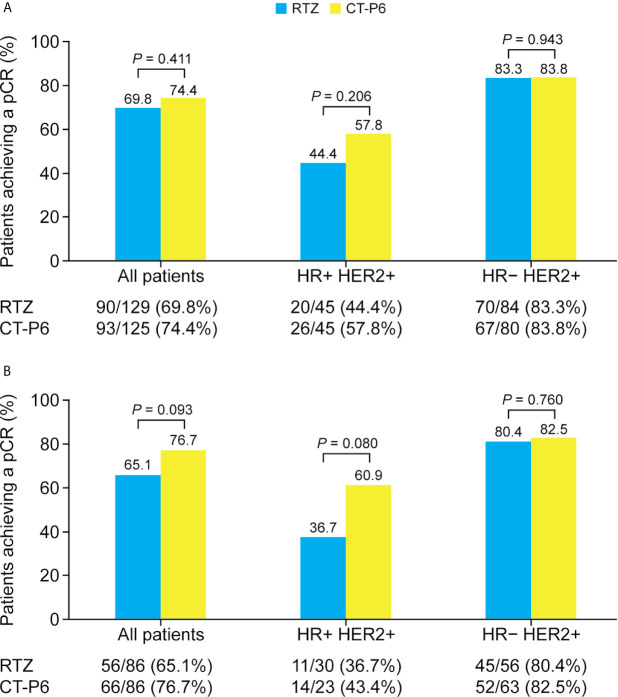
Percentage of patients with HER2-positive early-stage breast cancer who achieved a pCR following CT-P6 or RTZ treatment, stratified by HR status for **(A)** all patients and **(B)** propensity score matching cohort. HER2, human epidermal growth factor receptor 2; HR, hormone receptor; pCR, pathologic complete response; RTZ, reference trastuzumab.

The percentage of EBC patients who achieved a pCR did not differ between two groups (**Figure 2A**). Overall, 74.4% (93/125) achieved a pCR with CT-P6 versus 69.8% (90/129) with RTZ (*P*=0.411). Among patients with HR-positive EBC, 57.8% (26/45) of patients who received CT-P6 achieved a pCR, compared with 44.4% (20/45) of patients who received RTZ (*P*=0.206). Among patients with HR-negative, 83.8% (67/80) of patients who received CT-P6 achieved a pCR, as did 83.3% (70/84) of patients who received RTZ (*P*=0.943). Similarly, there was no difference in pCR between the two groups in the PSM cohort, regardless of subtype (**Figure 2B**).

**Figure 3:**
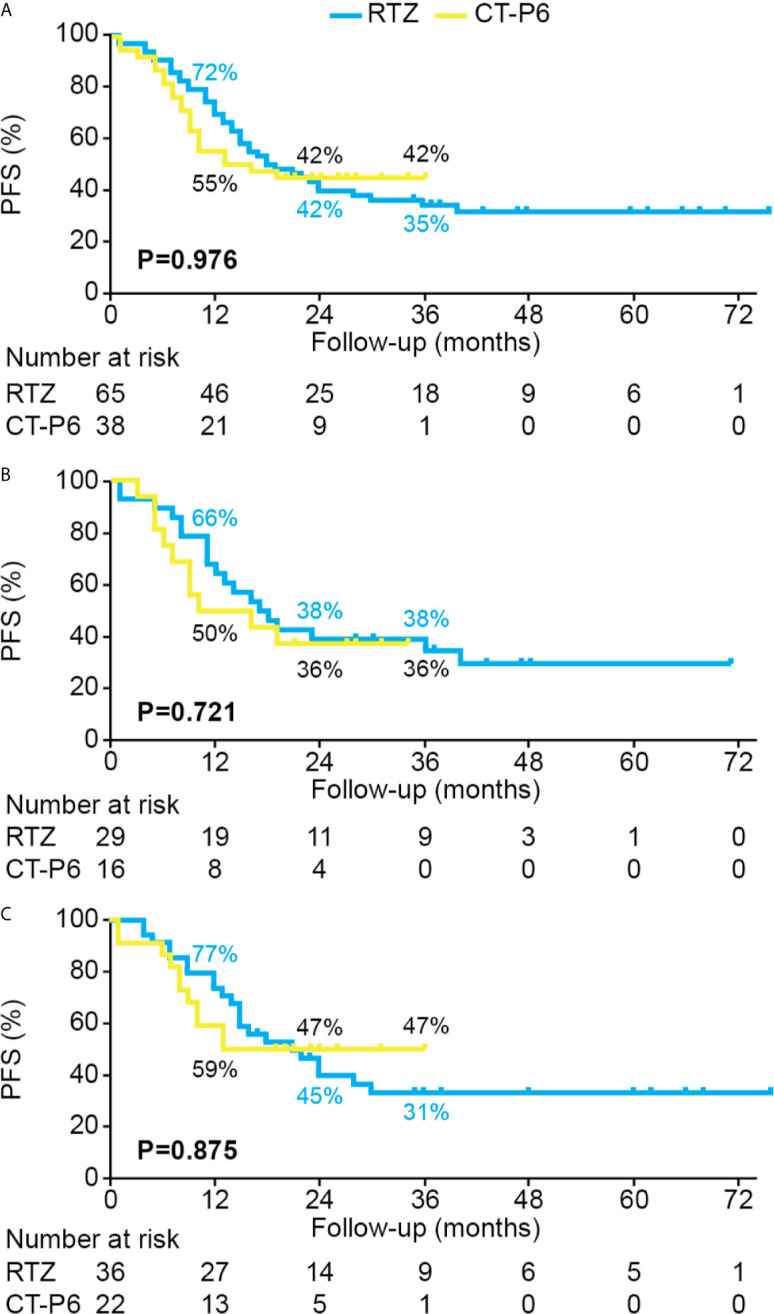
PFS for HER2-positive metastatic breast cancer patients who received palliative treatment with CT-P6 or RTZ for **(A)** all patients, **(B)** HR+/HER2+ patients, and **(C)** HR−/HER2+ patients. HER2, human epidermal growth factor receptor 2; HR, hormone receptor; PFS, progression-free survival; RTZ, reference trastuzumab.

The median follow-up time (range) was 23.0 (3.0-36.0) and 41.0 (1.0-83.0) months for MBC patients who received CT-P6 and RTZ. MBC patients in the CT-P6 received a median (range) of 23.0 (3–54) cycles while those in the RTZ received a median (range) of 26.0 (1-99) cycles. Median PFS did not differ significantly between two groups (CT-P6 vs RTZ: 13.0 vs 18.0 months [95% CI 0.0-26.6 vs 11.3-24.7]; *P*=0.976) (**Figure 3A**). There was also no statistically significant difference in median PFS between the two treatment groups regardless of HR status (HR-positive: CT-P6 vs RTZ 10.0 vs 17.0 months [95% CI 0.0-23.7 vs 10.0-24.0], *P*=0.721; HR-negative: 13.0 vs 21.0 months [95% CI NA vs 12.9-29.1], *P*=0.875) (**Figures 3B, C**). There was also no significant difference in mOS between CT-P6 and RTZ groups (not reached vs not reached, *P*=0.330) and regardless of HR status (data not shown). The ORR and DCR did not differ significantly (ORR: CT-P6 vs RTZ 78.9% vs 83.1%, *P*=0.602; DCR: 94.7% vs 93.8%, *P*>0.999) and also according to HR status (**Table 2**).

**Table 2:** Best overall response, based on RECIST 1.1 criteria, according to palliative treatment in patients with HER2-positive metastatic breast cancer.

	All patients			HR+/HER2+			HR−/HER2+		
	RTZ (*n*=65)	CT-P6 (*n*=38)	*P*-value	RTZ (*n*=29)	CT-P6 (*n*=16)	*P*-value	RTZ (*n*=36)	CT-P6 (*n*=22)	*P*-value
Overall response			0.706^a^			0.902^a^			0.389^a^
CR, *n* (%)	5 (7.7)	2 (5.3)		3 (10.3)	1 (6.3)		2 (5.6)	1 (4.5)	
PR, *n* (%)	49 (75.4)	28 (73.7)		18 (62.1)	11 (68.8)		31 (86.1)	17 (77.3)	
SD, *n* (%)	7 (10.8)	6 (15.8)		5 (17.2)	4 (25.0)		2 (5.6)	2 (9.1)	
PD, *n* (%)	2 (3.1)	2 (5.3)		2 (6.9)	0		0	2 (9.1)	
NA, *n* (%)	2 (3.1)	0		1 (3.4)	0		1 (2.8)	0	
Overall response rate			0.602			>0.999^a^			0.409^a^
CR/PR, *n* (%)	54 (83.1)	30 (78.9)		21 (72.4)	12 (75.0)		33 (91.7)	18 (81.8)	
Disease control rate			>0.999^a^			0.542^a^			0.551^a^
CR/PR/SD, *n* (%)	61 (93.8)	36 (94.7)		26 (89.7)	16 (100.0)		35 (97.2)	20 (90.9)	

a Fisher’s exact test.

CR, complete response; HER2, human epidermal growth factor receptor 2; HR, hormone receptor; NA, not available; PD, progressive disease; PR, partial response; RECIST 1.1, Response Evaluation Criteria in Solid Tumors version 1.1; RTZ, reference trastuzumab; SD, stable disease.

### Cardiac Safety of EBC and MBC Cohorts

In EBC cohort, the rate of LVEF did not differ between the two treatment groups before (mean [95% CI] LVEF: CT-P6: 68.1% [67.0–69.1] versus RTZ: 68.1% [67.1–69.1]; *P*=0.983) or after neoadjuvant chemotherapy (CT-P6: 65.9% [65.0–66.8] versus RTZ: 66.5% [65.3–67.7]; *P*=0.424) (**Supplementary Figure 1**). After neoadjuvant chemotherapy, 23/104 (22.1%) patients showed a decrease in LVEF of ≥10 percentage points from baseline in the CT-P6, compared with 13/90 (14.4%) patients in the RTZ (*P*=0.171). No patients in either treatment group showed a decrease in LVEF to <50% at any point.

In MBC cohort, both CT-P6 and RTZ showed a manageable cardiac safety profile, assessed with LVEF (**Supplementary Figure 2**). Nineteen out of 38 (50.0%) patients in the CT-P6 experienced a decline in LVEF of ≥10 percentage points from baseline, compared with 27/63 (42.9%) patients in the RTZ (*P*=0.695). Among those who received treatment with CT-P6, 3/38 (7.9%) patients experienced a reduction in LVEF to <50% at any time. One of these patients discontinued CT-P6 for 1 month before re-starting; the others continued treatment but later discontinued after 3 months owing to progressive disease. Among the patients who received treatment with RTZ, 4/65 (6.2%) experienced a reduction in LVEF to <50% at any time. Two of these patients discontinued treatment with RTZ, one discontinued for 1 month before re-starting, and the other continued treatment with RTZ.

## Discussion

To our knowledge, this is the first real-world study to show that CT-P6 has similar effectiveness and cardiac safety to RTZ in patients with HER2-positive EBC and MBC, when administered as part of dual HER2-targeted therapy with pertuzumab and chemotherapy in the neoadjuvant or palliative setting.

This study reported the similar pCR in the neoadjuvant setting for HER2-positive breast cancer with RTZ and CT-P6. Almost 75% of patients with HER2-positive EBC who received CT-P6 achieved a pCR, compared with around 70% of patients who received RTZ. In the EBC cohort, a significantly greater percentage of patients in the CT-P6 versus RTZ treatment group had a Ki67 index ≥14 at baseline (87.5% versus 70.5%; *P*=0.005). Notably, the Ki67 index is known to be a predictive marker for pCR in patients with breast cancer who receive neoadjuvant chemotherapy (24, 25). Therefore, we implemented PSM to reduce confounding bias: in the PSM cohort, there was no difference in response to treatment between the two groups, similar to the findings for the whole EBC cohort.

In the phase III randomized controlled trial that established the equivalence of CT-P6 and the reference product, no pertuzumab was added to the neoadjuvant regimen (16). However, since the dual HER2-targeted approach may further improve pCR rates compared with use of either RTZ alone (19, 23, 26, 27), most patients with HER2-positive breast cancer who received neoadjuvant chemotherapy at our institutions were treated with TCHP from 2018, when CT-P6 was approved. For this reason, we compared the efficacy of CT-P6 and RTZ among patients with HER2-positive EBC who received neoadjuvant TCHP. This study confirmed for the first time in routine clinical practice that CT-P6 is at least as effective as RTZ when administered as part of dual HER2-targeted therapy with pertuzumab.

This study also provided the first real-world evidence that CT-P6 is as effective as RTZ in patients with HER2-positive MBC, when administered with pertuzumab and docetaxel. In the CLEOPATRA trial, first-line treatment with improved PFS in patients with HER2-positive MBC compared with placebo-trastuzumab-docetaxel (18.5 vs 12.4 months; *P*<0.001) (23), and extended OS (56.5 vs 40.8 months; *P*<0.001) (21). A real-world study of patients with HER2-positive MBC in Italy observed a response rate of 77.3% with pertuzumab-trastuzumab-taxane, along with a median PFS of 21 months (28). These results are consistent with those of this study, in which the ORR was 78.9% with CT-P6-pertuzumab-docetaxel and 83.1% with RTZ-pertuzumab-docetaxel. There was also no significant difference in median PFS between patients who received CT-P6 or RTZ in this study (13.0 vs 18.0 months; *P*=0.976). While not statistically significant, the numerical difference in PFS between the treatment groups may reflect the shorter follow-up time for the CT-P6 group than RTZ (23.0 vs 41.0 months, respectively). However, since a relatively large number of censored events occurred early in the follow-up period for CT-P6, but later in the follow-up period for RTZ, a longer-term follow-up analysis will be required to confirm the equivalence of CT-P6 and RTZ with respect to median PFS.

Use of trastuzumab has historically been associated with an increased risk of cardiotoxicity (6), most often in the form of an asymptomatic decline in LVEF. In this study, neither CT-P6 nor RTZ showed serious cardiac toxicity profiles. No patients with EBC experienced a reduction in LVEF of <50% in either treatment group, consistent with the low cardiotoxicity observed in patients with EBC treated with RTZ or CT-P6 in the neoadjuvant (16, 20) and adjuvant (17, 20) settings. In this study, 7.9% (3/38) patients with MBC experienced a decline in LVEF to <50% with CT-P6, as did 6.2% (4/65) patients with RTZ. A similar decline in LVEF was observed in 6.1% of patients treated with pertuzumab-trastuzumab-docetaxel in the CLEOPATRA trial (21).

One strength of our study is that it included a broader patient population, that is more representative of the patient population as a whole than the highly selected individuals recruited into clinical trials. This is also the first study to compare the efficacy and safety of CT-P6 and RTZ in both EBC and MBC based on data from two institutions in Korea. There were also limitations: firstly, the study had a retrospective design and a relatively small sample size. Therefore, further prospective and larger studies are warranted. Secondly, the follow-up time for patients who received CT-P6 was shorter than for those who received RTZ. While the analysis found no significant difference in median PFS with CT-P6 versus RTZ, a longer follow-up period will be required to confirm their equivalence with respect to PFS, as well as OS.

Biosimilar drugs are a biological product that is highly similar to a licensed original product with clinical relevance in terms of safety, potency, and efficacy. The development of biosimilar drugs is necessary for reducing health costs and increasing global access to cancer treatment. Therefore, regulatory agencies [European Medicines Agency (EMA) and Food and Drug Administration (FDA)] have tried to approve drug development, change clinical practice and enter the market (29). Despite the established safety and efficacy profile of RTZ in HER2 positive breast cancer, the high cost of the drug remains a barrier to access, particularly in healthcare systems with fewer resources. For example, a study in China found that patients with HER2-positive EBC who lived in areas with a relatively high gross domestic product were more likely to receive RTZ than those in areas with a lower gross domestic product (30). The same study reported better survival outcomes in patients with EBC or MBC who received treatment with RTZ, illustrating how the high cost of RTZ can deprive patients of effective treatments. As in previous studies (8, 31, 32), the results of this study also suggest that biosimilar trastuzumab may provide treatment as safe and effective as RTZ, but at a remarkably presented opportunity for cost-saving and thereby to improved access for patients of HER2 positive breast cancer.This real-world study suggests that the biosimilar CT-P6 has similar effectiveness and cardiac safety to RTZ in HER2-positive EBC and MBC, when administered as part of dual HER2-targetd therapy with pertuzumab and chemotherapy in the neoadjuvant or palliative first-line setting. CT-P6 was well tolerated, with a cardiac safety profile similar to that of the RTZ. The increased use of biosimilars such as CT-P6 has the potential to boost access to life-extending treatments for women with HER2-positive breast cancer.

## Data Availability Statement

The raw data supporting the conclusions of this article will be made available by the authors, without undue reservation.

## Ethics Statement

The studies involving human participants were reviewed and approved by The institutional review boards (IRBs) of Yonsei Severance Hospital (IRB no. 4-2014-0551) and Gangnam Severance Hospital (IRB no. 3-2020-0090). Written informed consent for participation was not required for this study in accordance with the national legislation and the institutional requirements.

## Author Contributions

SB: Formal analysis, Investigation, Resources, Data curation, Writing - original draft, Visualization. JHK: Conceptualization, Methodology, Formal analysis, Investigation, Resources, Writing- original draft. SA: Conceptualization, Methodology, Investigation, Resources. H-CJ: Investigation, Resources. JS: Investigation, Resources. GK: Investigation, Resources. MK: Investigation, Resources. SK: Investigation, Resources. SP: Investigation, Resources, HP: Investigation, Resources, JYK: Investigation, Resources. JJ: Conceptualization, Methodology, Investigation, Resources, Writing - original draft, Writing – review and editing, Supervision, Project administration. All authors contributed to the article and approved the submitted version.

## Conflict of Interest

The authors declare that the research was conducted in the absence of any commercial or financial relationships that could be construed as a potential conflict of interest.
