# Supplementary material for: Real-World Clinical Outcomes of Biosimilar Trastuzumab (CT-P6) in HER2-Positive Early-Stage and Metastatic Breast Cancer
Source: Front Oncol. 2021 Jun 4;11:689587. doi: 10.3389/fonc.2021.689587 (PMC8213064; doi:10.3389/fonc.2021.689587)
Supplement: Supplementary file 1 [file DataSheet_1.docx]

**Supplementary Figure 1.** LVEF for patients with HER2-positive early-stage breast cancer before and after neoadjuvant treatment with CT-P6 or RTZ.

**
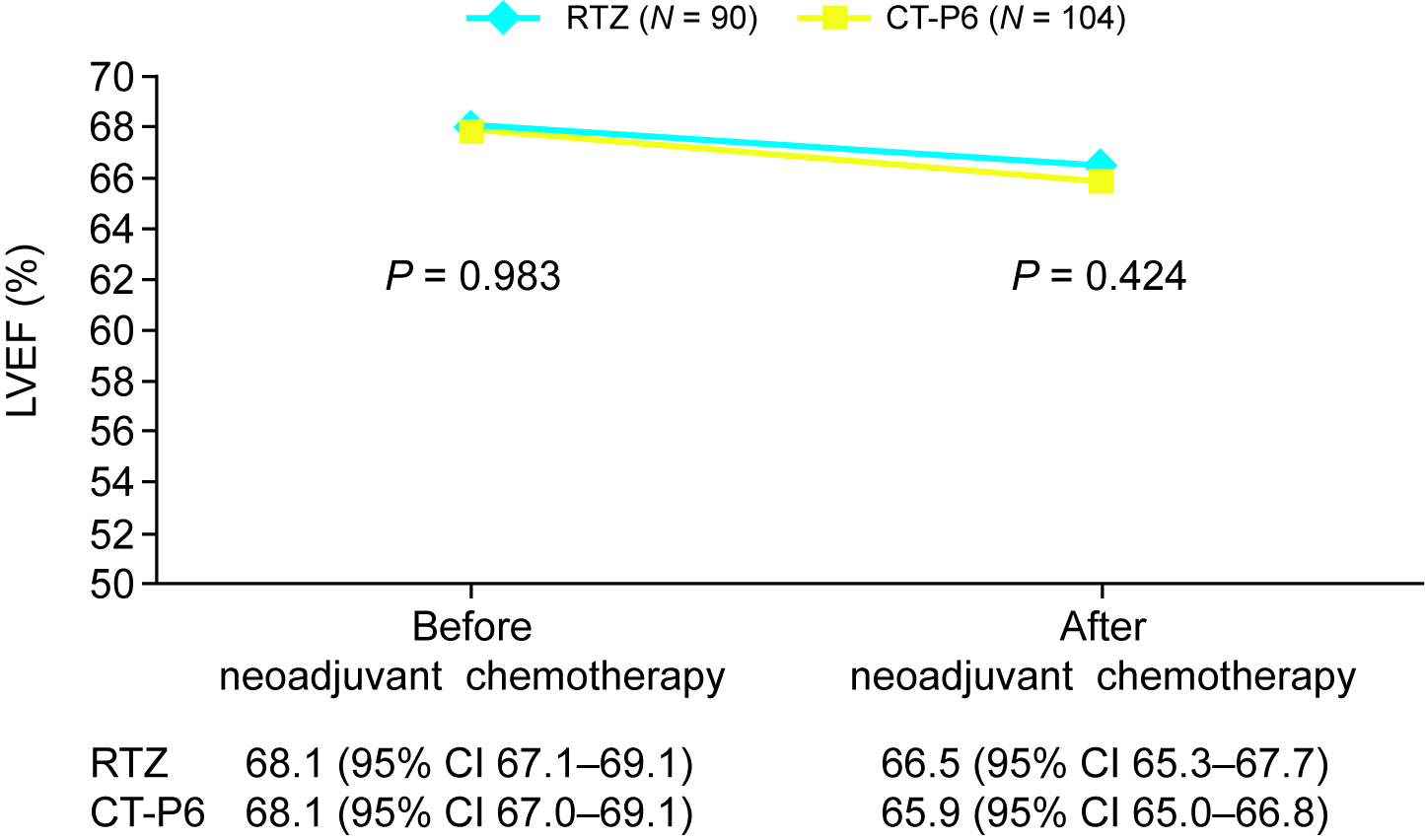
**

CI, confidence interval; HER2, human epidermal growth factor receptor 2; LVEF, left ventricular ejection fraction; RTZ, reference trastuzumab.

**Supplementary Figure 2** Change in LVEF over time for patients with HER2-positive metastatic breast cancer who received palliative treatment with CT-P6 or RTZ.

**
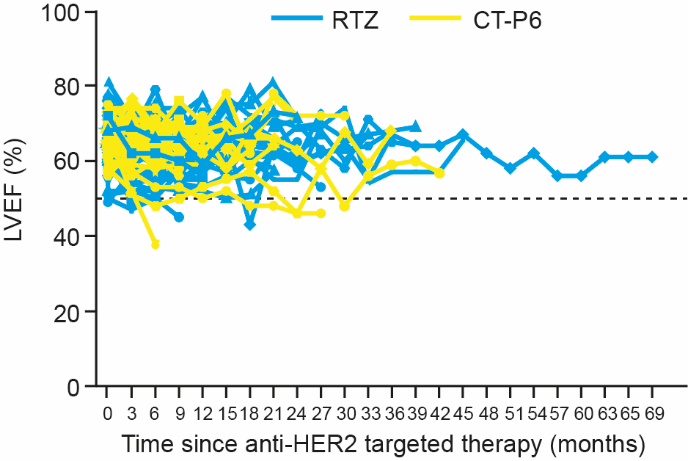
**

HER2, human epidermal growth factor receptor 2; LVEF, left ventricular ejection fraction; RTZ, reference trastuzumab.
