# Supplementary material for: Real-World Clinical Outcomes of Biosimilar Trastuzumab (CT-P6) in HER2-Positive Early-Stage and Metastatic Breast Cancer
Source: Front Oncol. 2021 Jun 4;11:689587. doi: 10.3389/fonc.2021.689587 (PMC8213064; doi:10.3389/fonc.2021.689587)
Supplement: Supplementary file 2 [file Table_1.docx]

**Supplementary Table 1.** Propensity score matching cohort (early-stage breast cancer).

|  | | **Early-stage breast cancer** | | | |
| --- | --- | --- | --- | --- | --- |
|  | | **RTZ (*n*=86)** | **CT-P6 (*n*=86)** | **Total (*N*=172)** | ***P*-value** |
| Age, median (range) | | 49 (27–71) | 51.0 (31–75) | 50 (27–75) | 0.497 |
| Histology, *n* (%) | |  |  |  | >0.999 |
|  | IDC | 84 (97.7) | 85 (98.8) | 169 (98.2) |  |
|  | ILC | 2 (2.3) | 1 (1.2) | 3 (1.7) |  |
| ER, *n* (%) | |  |  |  | 0.248 |
|  | Positive | 30 (34.9) | 23 (26.7) | 53 (30.8) |  |
|  | Negative | 56 (65.1) | 63 (73.3) | 119 (69.2) |  |
| PR, *n* (%) | |  |  |  | 0.708 |
|  | Positive | 19 (22.1) | 17(19.8) | 38 (20.9) |  |
|  | Negative | 67 (77.9) | 69 (80.3) | 136 (79.1) |  |
| Subgroup, *n* (%) | |  |  |  | 0.248 |
|  | HR+/HER2+ | 30 (34.9) | 23 (26.7) | 53 (30.8) |  |
|  | HR−/HER2+ | 56 (65.1) | 63 (73.3) | 119 (69.2) |  |
| HG,^a^ *n* (%) | |  |  |  | 0.173 |
|  | 1 or 2 | 59 (76.6) | 50 (66.7) | 109 (71.7) |  |
|  | 3 | 18 (23.4) | 25 (33.3) | 43 (28.3) |  |
| Ki67,^a^ *n* (%) | |  |  |  | 0.335 |
|  | <14 | 16 (27.1) | 12 (19.7) | 28 (23.3) |  |
|  | ≥14 | 43 (72.9) | 49 (80.3) | 92 (76.7) |  |
| cT,^b^ *n* (%) | |  |  |  | 0.742 |
|  | 1 or 2 | 58 (67.4) | 60 (69.8) | 118 (68.6) |  |
|  | ≥3 | 28 (32.6) | 26 (30.2) | 54 (31.4) |  |
| cN,^c^ *n* (%) | |  |  |  | 0.660 |
|  | Negative | 11 (12.8) | 13 (15.1) | 24 (13.9) |  |
|  | Positive | 75 (87.2) | 73 (84.9) | 148 (86.1) |  |

^a^Values were missing for some patients.

^b^cT was based on tumor size measured by pre-treatment breast magnetic resonance imaging.

^c^cN positive was defined as axillary lymph node metastasis proven by fine-needle aspiration biopsy (FNAB), or suspicious axillary lymph node metastasis in the imaging study among patients who did not receive FNAB.

cN, clinical node stage; cT, clinical tumor stage; ER, estrogen receptor; HER2, human epidermal growth factor receptor 2; HG, histologic grade; HR, hormone receptor; IDC, invasive ductal carcinoma; ILC, invasive lobular carcinoma; PR, progesterone receptor; RTZ, reference trastuzumab.
